# Dr. Martin

**Published:** 1879-08

**Authors:** E. T. Tappey

**Affiliations:** Berlin


### ARTICLE XII.

BERLIN, June 24th, 1879.

*To the Editors of the Chicago Medical Journal and Examiner, Chicago, Illinois:*

GENTLEMEN:—I have seen in your journal for April, 1879, an article entitled, "Miscellaneous Surgical Notes from Berlin," by Dr. Caldwell. In justice to Dr. Martin, I wish to make a few comments upon what Dr. Caldwell says upon the subject, "Amputation of the Cervix Uteri."

The doctor says: "I am sorry to be compelled to believe that, like some others of our noted gynæcologists, he (Dr. Martin) is bent on doing '*his*' operation as often as possible, and so submits many a poor woman to this treatment, *who might be relieved by a more simple procedure.*"

I do not object to the first part of the sentence, for naturally one's own operation must be the favorite one with its author, and he will perform it wherever he thinks it justifiable; this is especially true in the case of a new operation, where there are no theoretical objections to it, in order to produce statistics from which its efficacy can be judged. But I do object to the last clause, not only because I have sufficient confidence in Dr. Martin's honor to believe him incapable of such practice, but also because I do not believe this conclusion to be the result of reasonable judgment.

The object of this operation is, in many instances, not to cut away a diseased portion of the organ, but by its stimulating influence upon the tissues of the whole uterus, to cause involution of the same; and the operation may be very useful even when the objective symptoms are slight.

I believe that it is absolutely impossible that a simple frequenter of a gynæcological clinic, even if he does occasionally assist the operator, can be competent to judge of the necessity of an operation for the cure of chronic metritis (hyperplasia, as the condition is called by Dr. Thomas, of New York). Only the gynæcologist who has had experience, and who has carefully examined his patient — not once, but at intervals for a long time — can have a reasonable judgment of his own in the case.

Surely Dr. Caldwell does not fully realize the serious nature of the charge — for it amounts to a charge — he has brought against Dr. Martin in this careless manner.

Upon first reading that Dr. Caldwell has "seen him (Dr. Martin) and assisted him at this operation more than thirty times," one is inclined to believe that this is an experience upon which, surely, a reliable judgment can be founded; but, of course, each operation was performed upon a different patient, and no one of them was probably seen more than the ordinary clinical patient is seen.

As to the opinion that the accidents following his operations are kept in the background, I will simply say that it is a common practice of Dr. Martin to exhibit to his class the results of his operations at intervals, as long as the patients continue to be under his care.

Dr. Martin is not so "bent on doing '*his*' operation," but that I have seen him perform Schröder's and Hegar's and others, the authors of which I do not know; and he has told his class in my hearing, that the particular operation must depend upon the condition of the particular cervix uteri to be operated upon.

As to the case of occlusion of the os, following amputation, we all know that an accident or bad result is liable to follow any man's work, and that it is by our failures we learn more than by our successes. Now Dr. Martin places a rubber tube in the cervical canal, thus effectually preventing a repetition of such an accident.

In giving some statistics relating to this operation as performed by Dr. Martin, Dr. Caldwell says: "Of these cases only two died as a *direct result* of the operation," and in almost the next sentence tells us that one of these two "proved fatal from the supervention of typhoid fever." It seems to me extremely unfair to call the death of a typhoid fever patient a "direct result" of a surgical operation.

I have been a member of Dr. Martin's class for two months, and am so well pleased with his skill as an operator, and have such belief in his honor as a gentleman, that I think it a matter of simple justice to him to ask you to publish this in your journal.

Very respectfully yours,

E. T. TAPPEY, A.M., M.D.

---

LEAD POISONING FROM THE USE OF BABY-CARRIAGES.—According to the *Journal de Méd. de Bruxelles*, the covers of baby-carriages are constructed of a species of American leather, which contains a large quantity of lead, and, when exposed to the direct rays of the sun, they become a source of danger to the little innocents whom they are supposed to protect!

